# Cotton gauze wound-dressing patches loaded with dapsone derivative and silver nanoparticles for promoting wound healing *via* antibacterial acceleration function

**DOI:** 10.1039/d5ra04951b

**Published:** 2025-12-05

**Authors:** Huda R. M. Rashdan, Emad Tolba, Mohamed Abdelraof

**Affiliations:** a Chemistry of Natural and Microbial Products Department, Pharmaceutical and Drug Industries Research Institute, National Research Centre 33 El Buhouth St, Dokki Giza 12622 Egypt hudadawoud20@yahoo.com hr.rashdan@nrc.sci.eg; b Polymers and Pigments Department, National Research Centre 33 El-Bohouth St., Dokki, P.O. 12622 Giza Egypt; c Microbial Chemistry Department, Biotechnology Research Institute, National Research Centre Dokki Cairo 12622 Egypt

## Abstract

The development of multifunctional wound dressings with strong tissue adhesion, excellent bioactivity and effective antimicrobial properties is highly desirable for full-thickness skin wound healing. In this study, wound dressing patches were developed using a functionalized cotton dressing with a novel dapsone-based derivative (SBPBTT) and gelatin silver nanoparticles (AgNPs/Gel) for the rapid treatment of infected wounds. The structures of the AgNPs/Gel colloidal solution and modified cotton gauze were studied by electron microscopy. Both morphological observation and chemical compositions using electron microscopy (TEM/SEM) and X-ray photoelectron spectroscopy (XPS) of the treated modified cotton gauze confirm the integration of nanosized AgNPs (average particle size 124 ± 31 nm) onto the cotton fibers. The application of an AgNPs/SBPBTT^1^/gel coating significantly altered the properties of the cotton fabric. While untreated cotton was stable, the treated fabric exhibited a dramatic initial increase in hydrophilicity, with water uptake rising to ∼200%. This was accompanied by rapid biodegradation, with weight loss of up to 18.3% within 24 h. Our findings confirmed that the release rate of the prepared composite from the cotton gauze was an important factor to protect it from bacterial infection, when carried out at a suitable rate. In addition, an increase in composite concentration notably reduced potential infection by the tested bacterial pathogens. The ability of cotton-gauze-loaded material to serve as a bactericidal agent was also evaluated compared to reference antibiotics using standard microbial skin pathogens. Interestingly, a significant reduction in bacterial growth, particularly in the case of *Bacillus cereus*, followed by *Pseudomonas aeruginosa* and *Staphylococcus aureus*, was clearly dependent on the concentration and release rate of the loaded-AgNPs/gel.

## Abstract 2

The development of multifunctional wound dressings with strong tissue adhesion, excellent bioactivity and effective antimicrobial properties is highly desirable for full-thickness skin wound healing.

## Graphical abstract

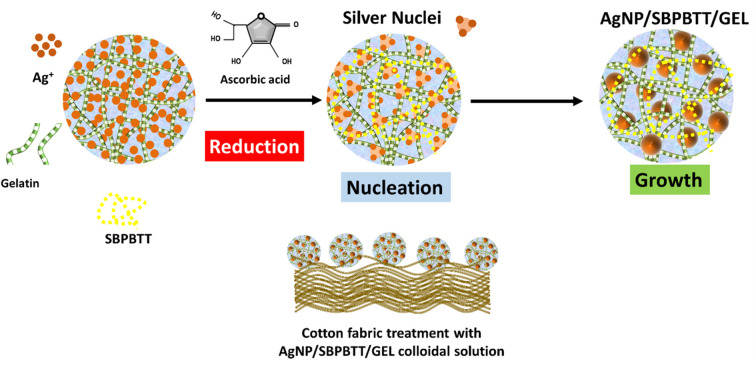

## 1 Introduction

Skin is the largest organ of the body, covering about 20 square feet. It performs numerous functions, including protecting against external elements like microbes, regulating body temperature, storing fluids and lipids, creating sensation with nerve endings that detect vibration, pressure, temperature, injury, and safeguarding muscles, bones and other vital organs. A skin wound is a pathogenic case resulting from any damage or disruption to skin tissues as a sudden result of direct chemical, mechanical or thermal trauma, or which develops slowly over a long time as a result of underlying disease processes like diabetes mellitus.^1–4^

Wound healing is a very complicated physiological process, involving interaction between various types of cells through different molecular mechanisms. When a wound fails to heal effectively or becomes a chronic wound, it causes severe pain and discomfort to patients. Consequently, investigation of novel treatment approaches can facilitate the wound-healing process and reduce the overall public health burden.^5^

Colonization by pathogenic microbes, along with the infectious bacteria present in the wound bed, poses a significant clinical barrier that hinders and degrades the physiological wound-healing process. The most common bacterial infections in wounds are caused by *Staphylococcus aureus*^6,7^ and *Pseudomonas aeruginosa* species. Moreover, the distribution of *Bacillus* species in wounds, although it is considered less aggressive than the most common bacterial species, causes skin inflammation, particularly in the case of *Bacillus cereus*. Compared to other microbes, *S. aureus* is mostly resistant to antibiotics and shows high virulence, contributing to the pathogenicity of the host. To address these challenges, innovative strategies are being developed to promote the wound–healing process.^8^

Recently, hemostatic agents have been developed in various forms, like hydrogels, sponges, powders and fabrics. Cotton gauze has been considered the preferred choice among these agents, owing to its affordability, breathability, durability, safety, lack of allergenicity, ability to manage traumatic bleeding and blood absorbency.^9–12^ Cotton gauze is usually applied during initial emergency care. Functionalizing cotton gauze involves modifying its surface or incorporating additional materials to enhance its properties for specific applications. This can be particularly beneficial in biomedical applications, where functionalized cotton gauze can be used for wound dressings, drug delivery, or antimicrobial purposes. Cotton gauze impregnated with therapeutic agents can serve as localized drug delivery systems, providing targeted treatment for various skin conditions and injuries. Over past decades, various bioactive agents, such as natural biomolecules (*e.g.*, flavonoids, vitamins, plant extracts, proteins, oils, polysaccharides)^13–15^ or synthetic materials (*e.g.*, gold, silver, zinc, titanium, copper and their oxides), have been employed for the surface treatment of cotton fabrics to provide antimicrobial and anti-inflammatory activities. Indeed, silver nanoparticles (AgNPs) are particularly effective bioactive substances because of their unique physicochemical properties and biological features. They exhibit superior antimicrobial activity due to their large surface area-to-volume ratio, which allows for greater interaction with the microbial cell membrane and even intracellular organelles.^16–18^ AgNPs can disrupt cell membranes, generate reactive oxygen species, and interfere with microbial DNA, leading to microbial cell death. These properties make AgNPs ideal for enhancing the microbial resistance of textiles used in medical applications.^19–21^

Dapsone (DAP) is an aniline-derived synthetic sulfonate widely used to treat several microbial infections like leprosy (also known as Hansen's disease, caused by *Mycobacterium leprae* and *Mycobacterium lepromatosi*s), acne, Kaposi's sarcoma and Behçet's disease; pneumocystis pneumonia (caused by *Pneumocystis jiroveci*, a yeast-like fungus) in various dermal conditions and in AIDS patients; epidermolysis bullosa acquisita; and systemic lupus erythematosus. DAP is also effective in treating malaria.^22–25^ The clinical impact of DAP was first investigated in 1945. It has been demonstrated that dapsone exhibits significant potent antioxidant, antiprotozoal, antimicrobial and anti-inflammatory efficacy. It also used as a wide-spectrum antibiotic.^26–29^ Additionally, DAP has the ability to manage the neutrophil inflammatory response. A neutrophil is a type of white blood cell that plays a core role in inflammatory responses, arriving first at the sites of damaged tissues to prevent microbial wound infections by killing the pathogenic microbes at the infected site.^30,31^ DAP also has the potency to prevent the generation of reactive oxygen species (ROS), decreasing the effect of eosinophil peroxidase on mast cells. It was also reported that DAP retards the production of toxic ROS. Moreover, DAP has the potency to inhibit elastase and myeloperoxidase (MPO).^32,33^ Furthermore, DAP can enhance the performance of monocytes and neutrophils to reduce oxidative stress.^34,35^

In this regard, a gel/AgNPs colloidal solution was synthesized through a green synthesis route and integrated into cotton gauze and loaded with a newly synthesized dapsone-based compound to develop a novel wound–healing patch. Additionally, the potency of the cotton-gauze-loaded material to serve as an antibacterial agent was also evaluated compared to reference antibiotics using standard microbial skin pathogenic microbes.

## 2 Materials and methods

### 2.1 Chemistry

#### 2.1.1 Raw materials

Dapsone, sodium azide, petroleum ether 40–60 °C, and absolute methanol were procured from Sigma-Aldrich. Bovine gelatin and silver nitrate (AgNO_3_) were purchased from Sigma-Aldrich Co. (Germany). The Textile Industry Company (El-Mahalla, Egypt) provided the cotton fabric, which weighed 147 g m^−2^. All the chemicals or solvents were used without further purification.

#### 2.1.2 Synthesis of 1,1′-((sulfonylbis(4,1-phenylene))bis(5-methyl-1*H*-1,2,3-triazole-1,4-diyl))bis(3-(thiophen-2-yl)prop-2-en-1-one) (SBPBTT)(3)

A mixture of 1-(1-(4-((4-(4-acetyl-5-methyl-1*H*-1,2,3-triazol-1-yl)phenyl)sulfonyl)phenyl)-5-methyl-4,5-dihydro-1*H*-1,2,3-triazol-4-yl)ethan-1-one (SBPTE) (4.66 g, 10 mmol), (10 mL, 10%) sodium hydroxide and thiophene-2-carbaldehyde (0.92 mL, 10 mmol) were ground thoroughly using a pestle in an open mortar for 5–15 min at room temperature until the mixture melted. Grinding was continued for approximately 5–15 min, and the reaction was monitored by TLC. The solid was collected and washed with water and then recrystallized from acetic acid to obtain the desired derivative 1,1′-((sulfonylbis(4,1-phenylene))bis(5-methyl-*1H*-1,2,3-triazole-1,4-diyl))bis(3-(thiophen-2-yl)prop-2-en-1-one) (SBPBTT) (3) as yellow crystals (92%); m.p. 210–212 °C, FT-IR (KBr, cm^−1^): *v* 1680 (C=O), 1622 (C=N), 1596 (C=C); ^1^H-NMR (DMSO-d_6_): *δ* 2.47 (s, 6H, 2CH_3_), 7.16 (d, 2H, *J* = 10.0 Hz, 2CH=C), 7.65–8.33 (m, 16H, ArH, 2CH=C) ppm; ^13^C-NMR (100 MHz, DMSO-d_6_): *δ* 9.90 (CH_3_), 126.62 (Ar), 128.83 (CH=C), 129.33 (Ar), 130.46 (Ar), 133.46 (CH=C), 136.02 (Ar), 139.17 (Ar), 139.25 (Ar), 141.48 (Ar), 143.12 (Ar), 182.61 (C=O) ppm; MS: *m*/*z* [%]: 652 (M); Anal. Calcd for C_32_H_24_N_6_O_4_S_3_ (652): C, C, 58.88; H, 3.71; N, 12.87%. Found C, 58.82; H, 3.66; N, 12.85%.

#### 2.1.3 Synthesis of silver/gelatin/SBPBTT colloidal solution

The synthesis of silver/gelatin/SBPBTT colloidal solution was carried out in a one-pot synthesis. In brief, the gelatin solution (as a stabilizing agent) was prepared by adding 3 g of gel to 50 mL of water under constant magnetic stirring at 40 °C. Then, 30 mL of freshly prepared AgNO_3_ (0.1 mM) was added to gel solution. The obtained gel/AgNO_3_ solution was kept at 40 °C away from light. Additionally, 10 mL of chloroform solution was mixed with a preset amount of SBPBTT, which was then sonicated for five minutes at 40 °C in an ultrasonic bath before being added to the AgNO_3_/gel solution. The obtained mixture was subject to vigorous stirring at 1000 rpm for ten minutes, and then 10 mL of ascorbic acid solution (0.1 M) was added gradually. The final solution changed from a yellowish to a yellowish brown color, indicating the formation of silver nanoparticles. To ensure the removal of chloroform, the reaction was kept under stirring for 10 h in the dark. SBPBTT was added at two different weight ratios relative to the gelatin content—5% (named AgNP/SBPBTT^1^/gel) and 10% (AgNP/SBPBTT^2^/gel).

#### 2.1.4 Treatment of cotton fabric with silver/gelatin/SBPBTT colloidal solution

The cotton fabrics were ultrasonically washed for 30 min at 40 °C in a 4% sodium lauryl sulfate solution. To maintain the temperature at 40 °C and counteract the heat generated by sonication, an ice bath was used as an external cooling system. The washed fabrics were then air-dried overnight at 40 °C. The dried fabrics were immersed in the obtained AgNP/SBPBTT/gel colloidal solutions and subjected to ultrasonic agitation for 1 h at 40 °C. An ice bath was used to dissipate heat from sonication and maintain a constant 40 °C, preventing thermal damage to sensitive compounds such as gelatin and SBPBTT. At elevated temperatures (typically above 40 °C, depending on the type), the protein chains can unfold (denature), destroying their gelling properties and altering their ability to stabilize the nanoparticles and adhere to the fabric.^36^ Following the sonication step, the cotton cloth was cleaned with wet filter paper and dried for 24 h at room temperature.

#### 2.1.5 Morphological and elemental characterization

Using various analytical methods, the morphology and elemental composition of the prepared samples were investigated. ^1^H and ^13^C-NMR spectra were recorded in (CD_3_)_2_SO solution on a BRUKER 500 FT-NMR spectrometer, and chemical shifts are expressed in ppm using TMS as an internal reference. Mass spectra were recorded on a Shimadzu GC-MS QP1000 EX. Elemental analyses were carried out at the Microanalytical Center of Cairo University. The absorption peak of the synthesized Ag NPs was observed with a UV-visible spectrophotometer (Shimadzu (UV-2500), Japan) in the range of 200–900 nm. Using an ATR technique and a JASCO FT/IR-460 spectrophotometer (Japan) to record Fourier transform-infrared (FTIR) spectra in the 400–4000 cm^−1^ range, the cationic and anionic alterations in the sample surface were investigated. Dynamic light scattering (DLS) measurements were performed using a Zetasizer Nano ZS90 System (Malvern Instruments) to provide critical information about the particle size distribution of the synthesized gel/AgNPs. TEM and SEM techniques were used to visualize the morphology and the size of the synthesized Ag NPs using an ultra-high-resolution transmission electron microscope (JEOL-2010). In addition, the cotton-treated samples were coated with gold and examined using a field emission scanning electron microscope (SEM) (Jeol JXA 840, Japan) and spatially resolved energy-dispersive X-ray spectroscopy (EDX, Jeol JXA 840, Japan). The elements of C, O, and Ag have been used to show the distribution of AgNPs on the cotton gauze. In addition, X-ray photoelectron spectroscopy (XPS) was performed on a K-ALPHA instrument (Thermo Fisher Scientific, USA) with monochromatic X-ray Al K-alpha radiation at −10 to 1350 eV with a spot size of around 400 µm at a pressure of ∼10 mbar and full-spectrum pass energy of 200 eV and at a narrow spectrum of 50 eV.

#### 2.1.6 Water uptake and weight loss

The neat cotton and AgNPs-treated cotton fabrics were cut into nearly equal-sized pieces (1 × 1 cm) and weighed using a digital balance. This weight was termed the initial weight (*W*_i_). Then they were immersed in 20 mL of phosphate buffer solution (PBS) and incubated at 37 °C in closed sterilized plastic tubes for 1, 2, 4, 5 and 6 days. After each prescribed time, the wet samples were taken out and put on filter paper to absorb all the surface solution, and were then weighed, denoted the wet weight (*W*_w_). After drying for 24 h, the samples were weighed again and termed the dry weight (*W*_d_). The weight loss and the water uptake percentages were calculated according to the following relations:

1 Weight loss (%) = [(*W*_d_ − *W*_i_)/*W*_i_] × 100

2 Water uptake (%) = [(*W*_w_ − *W*_i_)/*W*_i_] × 100

### 2.2 Antimicrobial activity

#### 2.2.1 Investigation of the antibacterial susceptibility of the synthesized molecules

The efficiency of the prepared cotton gauze materials (cotton gauze and AgNP/SBPBTT^2^/gel-treated cotton gauze) to prevent the survival of bacterial-wound pathogens was investigated *in vitro*. In this regard, very important skin wound infection pathogens were used as models, such as *Pseudomonas aeruginosa*, *Bacillus cereus* and methicillin-resistant *Staphylococcus aureus* (MRSA). In acute and chronic wounds, *Pseudomonas aeruginosa* and MRSA were usually found to be outnumbered by other bacterial flora. Therefore, the selection of these bacterial types supported our goals in this study, and they were kindly donated by the Microbiology and Immunology Department, Faculty of Medicine, Al-Azhar University. Pre-activation of bacterial pathogens was performed in Mueller–Hinton broth culture medium for 24 h at 35 °C under shaking. After confirmation of the inoculum size for each bacterial pathogen using serial dilution, which became relatively constant at 10^−6^, the agar well diffusion procedure was used to screen all samples. For this purpose, each material was prepared with constant dimensions, and starter molecules were also prepared at 20 µg mL^−1^ using DMSO reagent. After pouring Mueller–Hinton agar medium, each bacterial pathogen was added with a sterilized swab, and a 3 mm hole was made in the agar to add the starter compounds, while the cotton material was added using sterilized forceps. The antibacterial activity of each of the tested materials and starter molecules was measured according to the diameter (mm) of the inhibition zone of the bacterial pathogen around each tested sample at the end of the incubation time.^37^ To avoid the cotton material hindering diffusion, another method was also applied using turbidometric and colony-forming unit (CFU) procedures in the MHB medium.^38^ Furthermore, cephalexin (CL-30), ciprofloxacin (CI-30), ampicillin (AP-20), amoxicillin (AX-25), polymyxin B 300 U (PB-300), flucloxacillin (FL-5), rifamycin (RF-30), neomycin (N-30), clindamycin (DA-2), piperacillin + tazobactam (TPZ-110), and erythromycin (E-15) were used as positive control agents.^39^

#### 2.2.2 Determination of minimum inhibition concentration (MIC) of the most-active cotton-gauze-loaded material

In this experiment, each starter compound was subjected to the determination of the MIC value using different concentrations of each. In this way, each starter compound was justified by a potent MIC to incorporate it into the material and then to investigate its capability to inhibit a great number of bacterial colonies. The MIC value was determined using the microdilution method. The MIC was defined as the lowest concentration of each material sample at which the number of colony-forming units (CFU) was reduced compared to the untreated samples.^40^

#### 2.2.3 Measurement of intracellular reactive oxygen species (ROS) inside the treated bacterial cells

Reactive oxygen species are assumed to be involved in mechanisms that may be responsible for the antibacterial activity of sulfa drugs. ROS are known as a group of highly reactive molecules, like molecular oxygen (O_2_), superoxide anion (O_2_˙^−^), hydrogen peroxide (H_2_O_2_) and hydroxyl radicals (˙OH), with the ability to induce oxidative stress in bacterial cells. Therefore, the ability of the tested cotton materials to induce ROS toward bacterial pathogens was assessed intracellularly. In this way, ROS could be quantified using a fluorescent probe, 2′,7′-dichlorodihydrofluorescein diacetate (DCFH-DA). Briefly, the treated bacterial samples were subjected to a permeabilization process by ultrasonication of the cells, and the resultant supernatant was harvested and reacted with 10 µM DCFH-DA for 30 min at 37 °C.^37^ At the same time, the bacterial cells were treated with hydrogen peroxide H_2_O_2_ at 155 µM as a positive control. The DCF fluorescence intensity was measured immediately using spectrofluorometric apparatus (JASCO FP-6500, light source xenon arc lamp, Japan). The fluorescence intensity for each sample was measured with excitation and emission wavelengths of 485 nm and 530 nm, respectively.

#### 2.2.4 Effect of lipid peroxidation (LPO)

Oxidation of the fatty acid content of a bacterial cell membrane is considered a significant marker of oxidative stress on bacterial cells. The lipid peroxidation process triggers the by-product malondialdehyde (MDA), which reacts with a specific reagent, thiobarbituric acid (TBA), to form an MDA-TBA complex (pink color), and can be identified using a lipid peroxidation MDA assay kit.^41^ After determining the optimal concentration of promising compounds, treatment of each bacterial pathogen was carried out under standard conditions for 24 h under shaking. Subsequently, 300 µL of MDA lysis buffer was vigorously homogenized with 1 mL of the bacterial suspension on ice. To overcome interference from pigments formed during the degradation of lipophilic peroxides and to target the true pigments formed during oxidative stress, 3 µL of butylated hydroxytoluene (BHT) was added. Centrifugation of the samples at 8000 rpm for 10 min was conducted to remove insoluble materials. After that, 200 µL of the supernatant of each treated sample and 600 µL of TBA solution were mixed at 95 °C for one hour.^42^ Samples were then cooled for 10 min, and the formed MDA-TBA complex was measured at 532 nm using a spectrophotometer (Agilent Cary 100, Germany). In addition, a positive control was also prepared using hydrogen peroxide at 5% concentration, and the bacterial cells were treated for 20 min. The percentage increase in efficiency (%) of lipid peroxidation for the treated bacterial cells was calculated as follows:

100 [(NS − NC)/NC] ×

where NC is the absorbance from lipid peroxidation in the untreated bacterial cells, and NS is the absorbance from lipid peroxidation in the treated bacterial cells.

#### 2.2.5 Cytotoxic evaluation

The cytotoxic effect of the tested samples on fibroblast cells (obtained from VACSERA, Egypt) was assessed using an MTT assay (3-(4,5-dimethylthiazol-2-yl)-2,5-diphenyltetrazolium bromide). Fibroblast cells (1 × 10^4^ cells/well) were seeded in 96-well plates and incubated overnight at 37 °C in a humidified 5% CO_2_ atmosphere. On the following day, the culture medium was replaced, and the cells were treated with increasing concentrations (50, 25, and 10 µg of the newly synthesized tested samples) for 24 h. After treatment, 10 µL of MTT solution (5 mg mL^−1^ in PBS; Sigma-Aldrich, USA) was added to each well and incubated for 4 h at 37 °C under dark conditions. The resulting formazan crystals were dissolved by adding 100 µL of dimethyl sulfoxide (DMSO; Sigma-Aldrich, USA) per well and shaking for 5 min. Absorbance was measured at 570 nm using a BioTek microplate reader (Agilent Technologies, USA). Cell viability (%) was calculated using the formula [1 − (ODt/ODc) × 100], where ODt is the mean optical density of treated wells, and ODc is that of the control wells. Each concentration was tested in triplicate, and three independent biological replicates were performed. The IC_50_ value was determined by nonlinear regression analysis (GraphPad Prism).^43^

## 3 Results and discussions

### 3.1 Chemistry

**Scheme 1:**
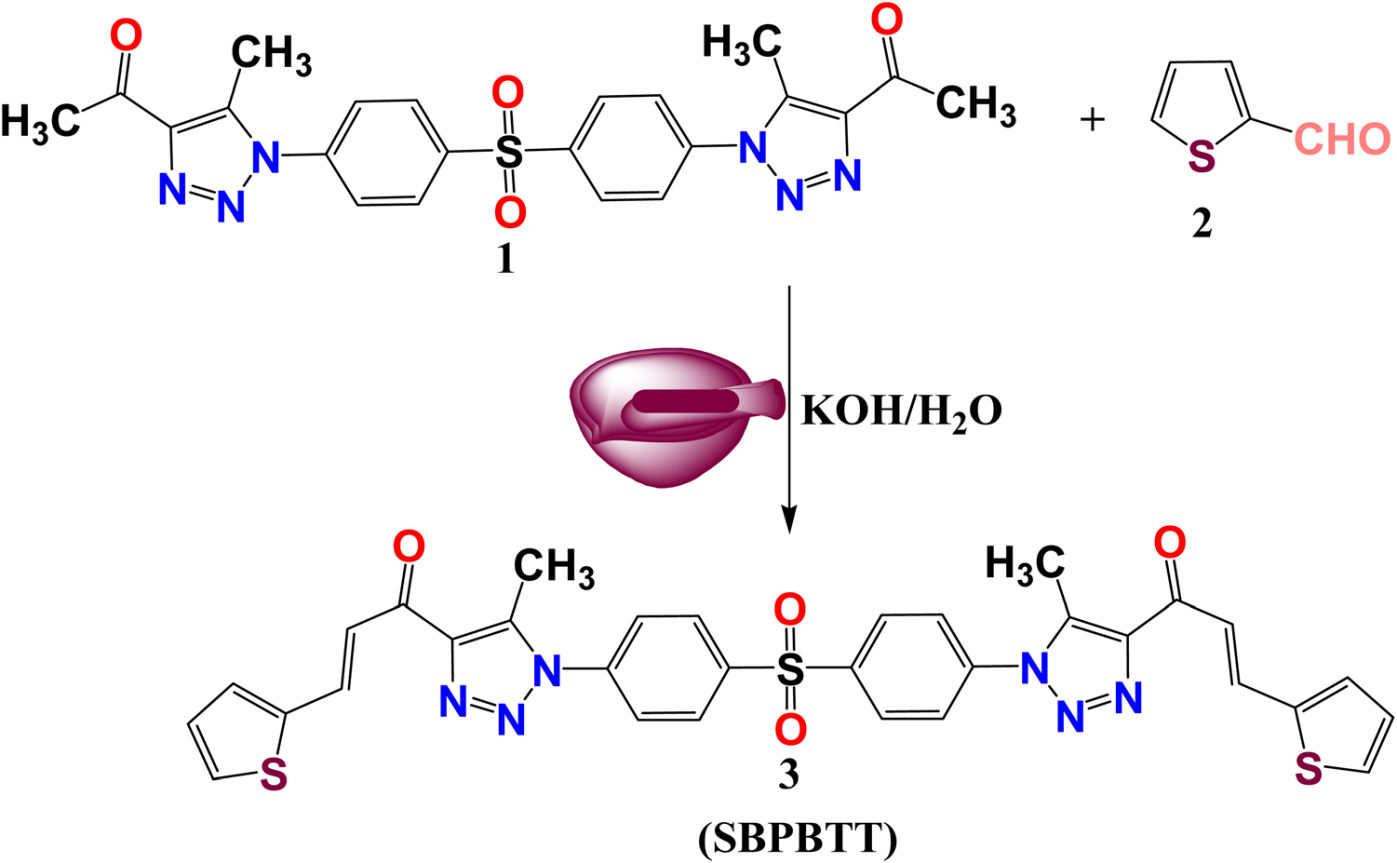
Synthesis procedures of 1,1′-((sulfonylbis(4,1-phenylene))bis(5-methyl-1*H*-1,2,3-triazole-1,4-diyl))bis(3-(thiophen-2-yl)prop-2-en-1-one) (SBPBTT) (3).

**Fig. 1:**
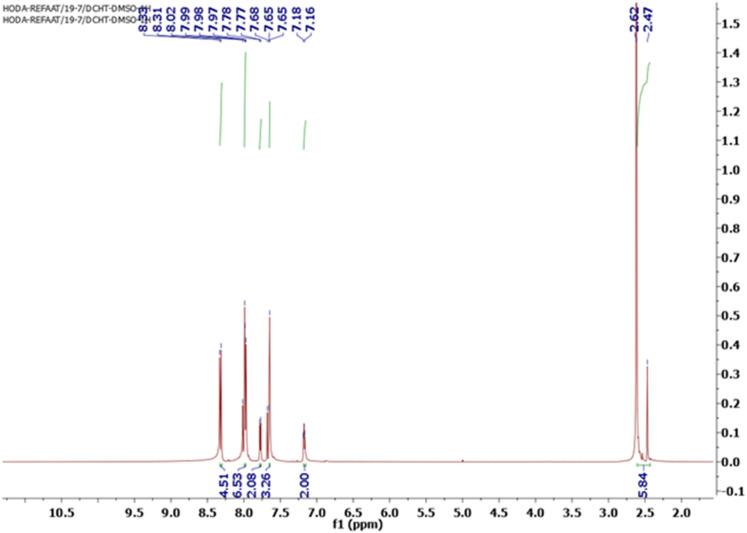
H^1^-NMR spectrum of 1,1′-((sulfonylbis(4,1-phenylene))bis(5-methyl-1*H*-1,2,3-triazole-1,4-diyl))bis(3-(thiophen-2-yl)prop-2-en-1-one) (SBPBTT) (3).

**Fig. 2:**
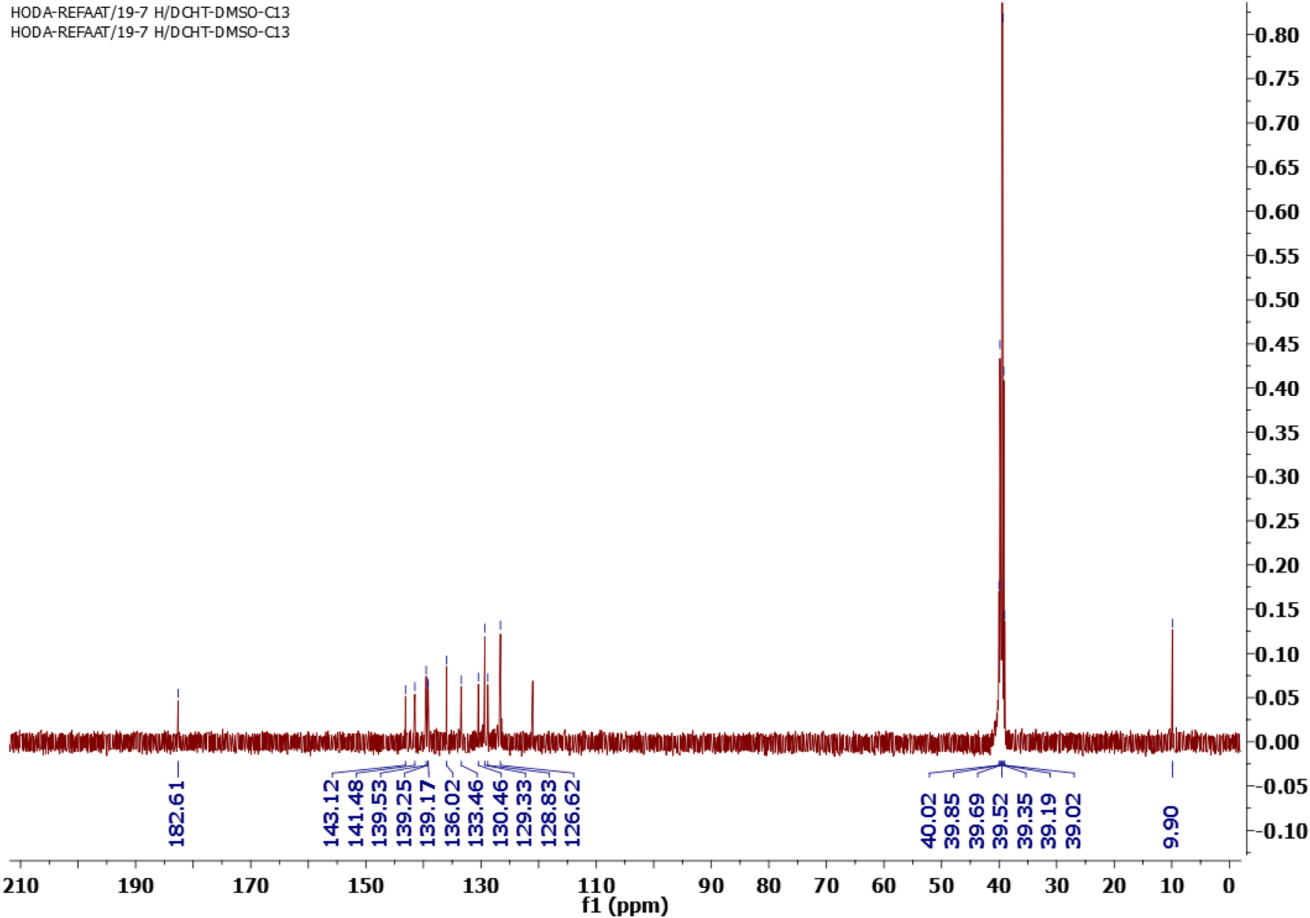
C^13^-NMR spectrum of 1,1′-((sulfonylbis(4,1-phenylene))bis(5-methyl-1*H*-1,2,3-triazole-1,4-diyl))bis(3-(thiophen-2-yl)prop-2-en-1-one) (SBPBTT) (3).

Owing to the variability in side chains and the associated charges of the chalcone derivatives, which introduce the viability of binding to different proteins involved in essential biological functions, the chalcone compounds may have different pharmaceutical and biomedical impacts. In order to synthesize new chalcone derivatives based on the sulfone bis-compound, the Claisen–Schmidt condensation method was utilized. Additionally, a solvent-free protocol was applied as a green chemistry approach *via* grinding the solids/liquids together using a mortar and pestle at room temperature, in which a mixture of 1-(1-(4-((4-(4-acetyl-5-methyl-1*H*-1,2,3-triazol-1-yl)phenyl)sulfonyl)phenyl)-5-methyl-4,5-dihydro-1*H*-1,2,3-triazol-4-yl)ethan-1-one (SBPTE) (1), 10% aqueous potassium hydroxide and thiophene-2-carbaldehyde was ground thoroughly together to afford the corresponding desired compound 1,1′-((sulfonylbis(4,1-phenylene))bis(5-methyl-1*H*-1,2,3-triazole-1,4-diyl))bis(3-(thiophen-2-yl)prop-2-en-1-one) (SBPBTT) (3) in an excellent yield of approximately 92% (Scheme 1). The chemical structure of the SBPBTT was characterized and confirmed from the correct spectral and microanalytical data by employing ^1^H and ^13^C-NMR and mass spectroscopic analyses. The formation of the carbon–carbon double bond results in the production of the desired chalcone derivatives in either Z or E configuration. Vinyl protons appeared as doublets between 7.16 and 7.77 ppm with a coupling constant of *J* = 10.0 Hz (Fig. 1). Meanwhile, the vinyl protons were recorded in the C^13^-NMR spectrum at 128.83 and 133.46 ppm (Fig. 2). The structure was also supported by its mass spectrum MS *m*/*z* (%): 652, which agrees with its molecular formula C_32_H_24_N_6_O_4_S_3_.

### 3.2 Treatment of cotton fabric with silver/gelatin/SBPBTT

**Fig. 3:**
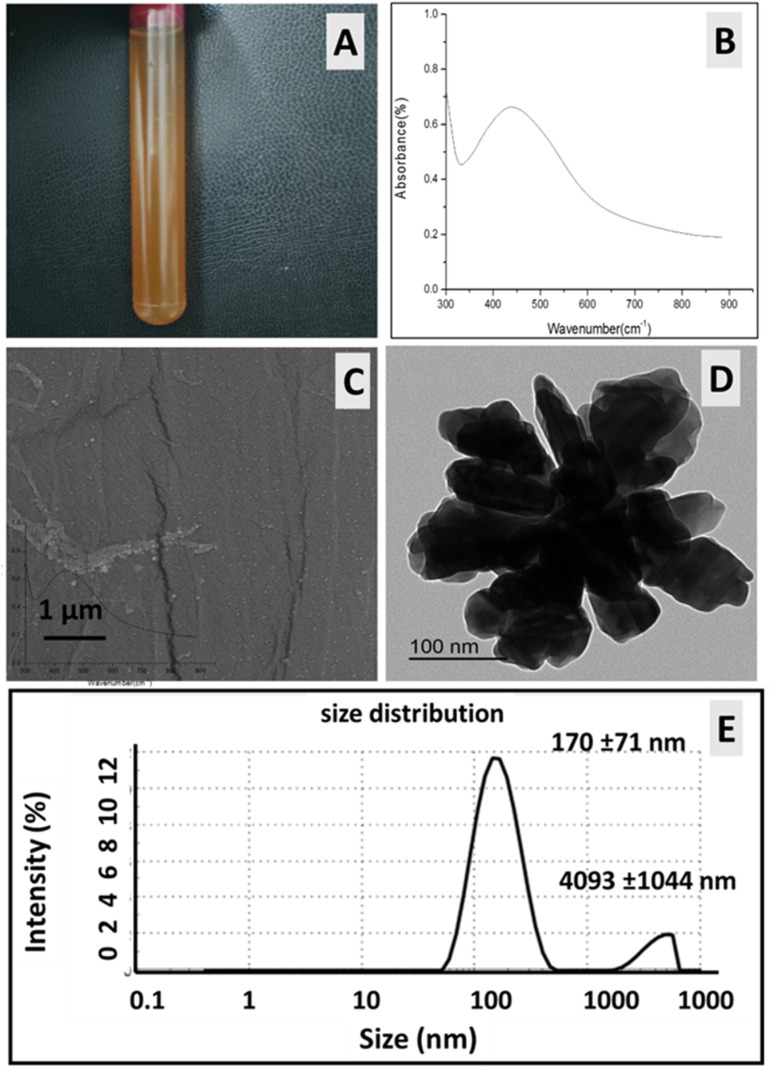
Characterization of the as-prepared AgNPs colloidal solutions: (A) color change of AgNPs/gel solution after the addition of ascorbic acid; (B) UV-visible spectrophotometer range from 300 to 900 nm; (C) SEM image of AgNPs/gel; (D) TEM image of AgNPs/gel colloidal solution and (E) AgNPs particle size distribution based on DLS measurements.

In this study, gel was used to stabilize the formed AgNPs and to improve their integration into the cotton fabric. After the reduction process, it was observed that the colour of the gel solution changed to brown, as shown in Fig. 3A. The formation of AgNPs within the gel matrix was confirmed by the existence of a single surface plasmon resonance (SPR) peak in the UV-vis absorption spectrum (Fig. 3B), due to AgNPs. The UV-vis spectrum shows strong absorbance at a wavelength of 445 nm, as demonstrated in Fig. 3B. TEM and SEM of the obtained AgNP/gel colodial soltion are shown in Fig. 3C and D. As can be seen in both images, the AgNPs presented oval- and triangle-shaped nanoparticles with an average length of 124 ± 31 nm and width of 74 ± 31. In addition, the DLS analysis showed that the mean size distribution was 170 ± 71 nm and the PDI value was 0.54 (Fig. 3E), confirming the size calculated from the TEM analysis (Fig. 3C). The results also indicate a very slight degree of aggregation, resulting in a small peak shoulder at 4093 ± 1044 nm.

**Fig. 4:**
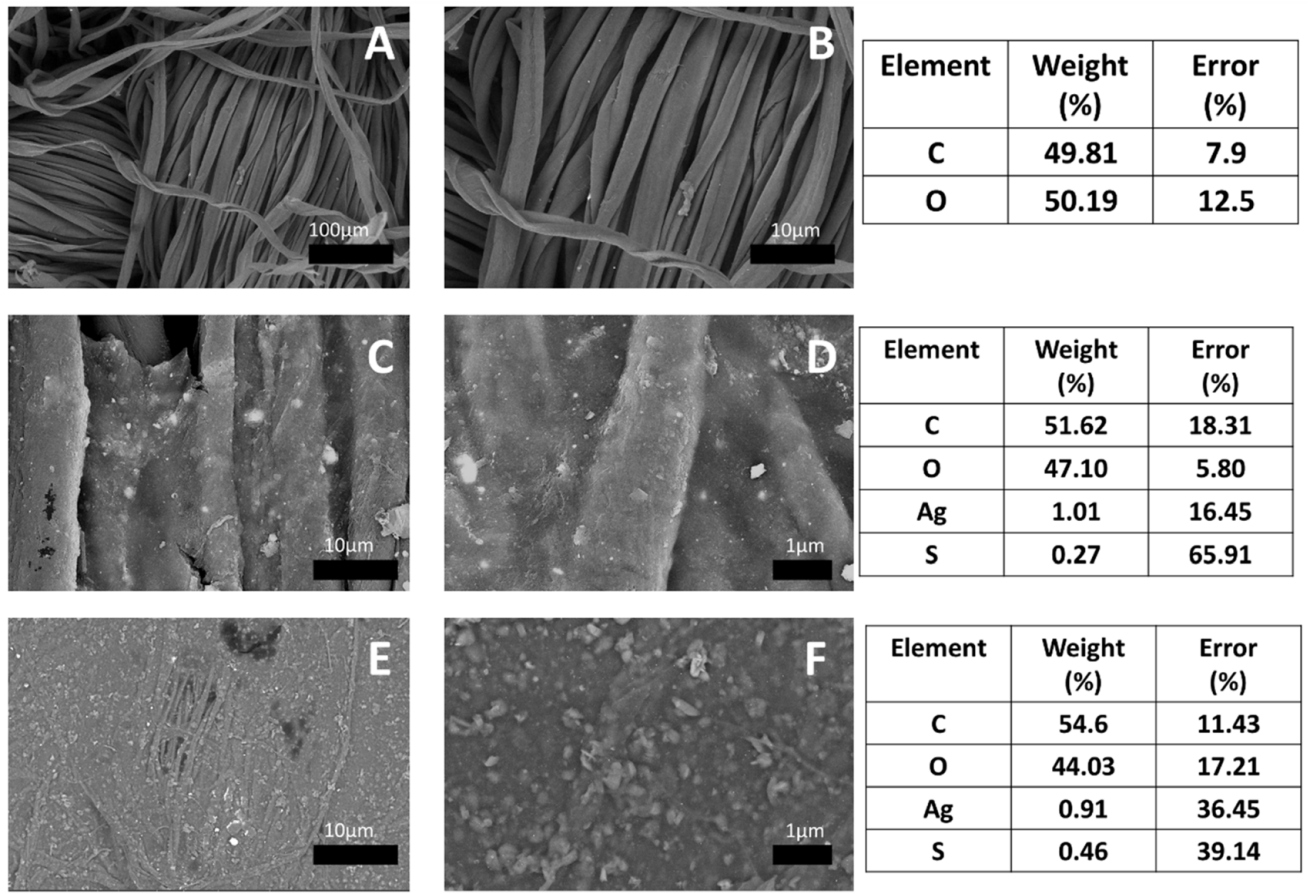
SEM micrographs of (A and B) untreated cotton gauze (control), (C and D) AgNP/SBPBTT^1^/gel-treated cotton gauze and (E and F)AgNP/SBPBTT^2^/gel-treated cotton gauze samples.

SEM analysis indicates the formation of a gelation layer on the cotton gauze surface. The untreated cotton gauze (blank) had a woven fiber structure (Fig. 4A and B). While the treated samples showed a continuous layer on the surface of cotton fibers with a rough surface, which could be due to the embedded AgNPs and SBPBTT, as shown in Fig. 4C–F. In addition, Ag and sulfur elements were detected on the surface of the cotton treated with AgNPs/SBPBTT^1,2^/gel samples. The sulfur content was higher in cotton gauze treated with AgNPs/SBPBTT^2^/gel than with AgNPs/SBPBTT^1^/gel colloidal solution due to the increase in SBPBTT concentration from 5 to 10 wt%.

**Fig. 5:**
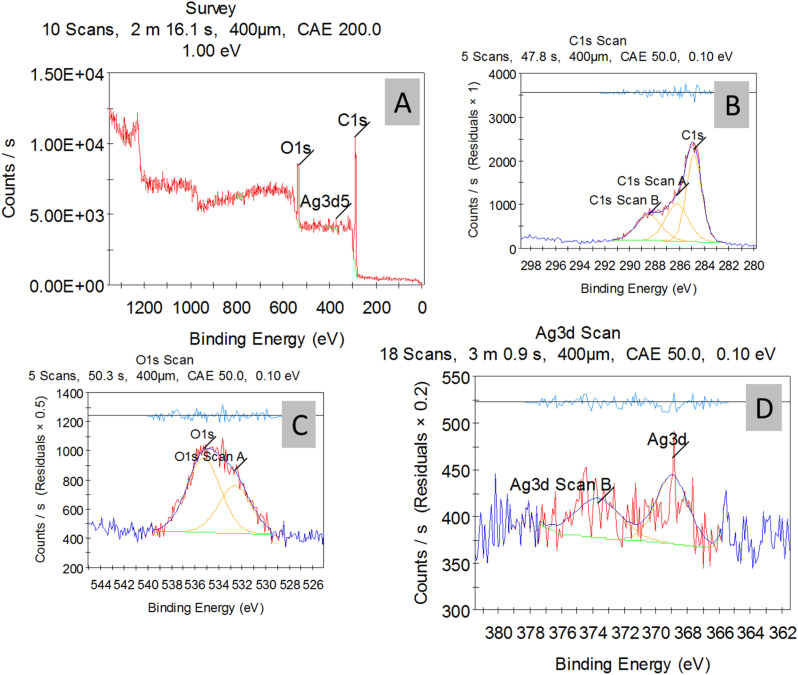
XPS spectrum of the AgNP/SBPBTT^2^/gel-treated cotton gauze sample (A); high-resolution C 1s scan (B); high-resolution O 1s scan; (C) and high-resolution Ag 3d 5 scan (D).

The chemical structure of the AgNPs-treated cotton fabric was analyzed by XPS. The wide-scan spectrum of the AgNP/SBPBTT^2^/gel-treated cotton gauze sample (Fig. 5A) exhibited distinct photoelectron peaks for carbon (C 1s), oxygen (O 1s), and silver (Ag 3d). The presence of C 1s and O 1s is to be expected (Fig. 5B and C). They originate from the cellulose of the cotton fabric itself (which is made of glucose units, C_6_H_10_O_5_) and from the composite coating (SBPBTT^2^ and gel, which are organic biopolymers rich in carbon and oxygen). Deconvolution of the C 1s spectrum (Fig. 5B) resolved it into three constituent peaks, which were assigned to C–C/C–N, C–OH, and C–O–C moieties. Simultaneously, the Ag 3d spectrum (Fig. 5D) showed peaks at 368.0 eV (Ag 3d_5/2_) and 374.0 eV (Ag 3d_3/2_), which are definitive signatures of silver in its metallic state (Ag^0^), confirming the formation of silver nanoparticles within the composite coating.^44,45^

### 3.3 Antimicrobial screening

**Fig. 6:**
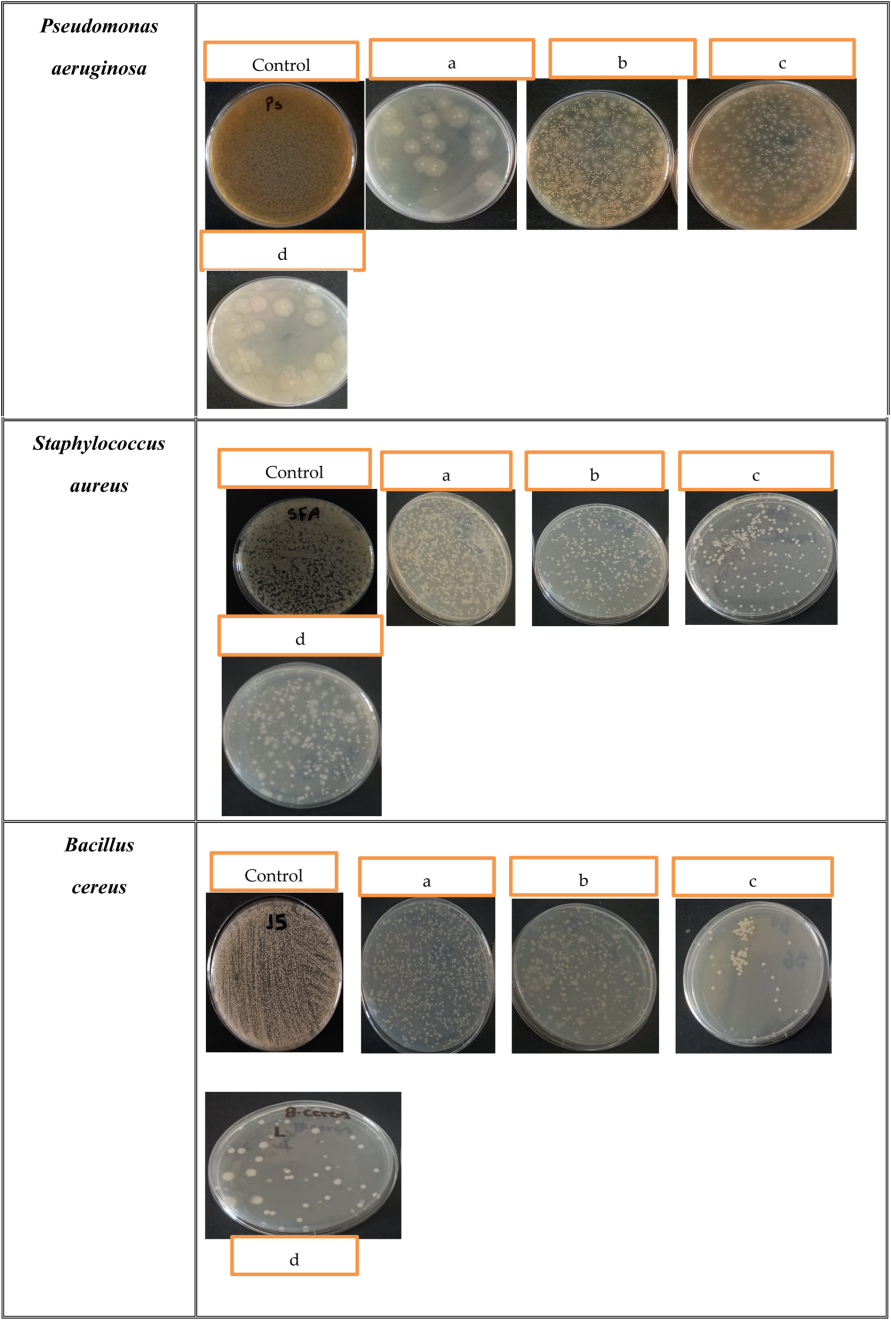
Antibacterial activity of the targeted materials against *P. aeruginosa*, *S. aureus* and *B. cereus* using the colony-forming unit (CFU) method. Each bacterial test was incubated in the presence of the target material for 24 h. (a) Cotton gauze with (AgNPs/gel); (b) cotton gauze with AgNPs/SBPBTT^1^/gel; (c) cotton gauze with AgNPs/SBPBTT^2^/gel; (d) cotton gauze with AgNPs.

As shown in Table 1 and Fig. 6, different responses by each of the cotton-gauze-loaded materials toward each bacterial pathogen were clearly indicated. During incubation with each bacterial pathogen (in the presence of each tested material), the turbidity and CFU counts were measured after treatment. The SBPBTT sample showed a marked increase in antibacterial activity, highlighting its strong suppressive action on bacteria due to the higher concentration of released AgNPs/SBPBTT/gel compared to other materials under the same conditions. Thus, potential eradication of bacterial pathogens was obviously implemented in the case of compound SBPBTT; however, the inhibition ratio for other materials was either negligible or slightly inhibited for samples 1 and 2. In particular, the survival of *Bacillus cereus* was significantly more affected when compared to other tested bacteria, which could be correlated with the sensitivity of this bacterium to the released AgNPs/SBPBTT/gel. *Pseudomonas aeruginosa* was affected by the released composite, and more than 50% of its proliferative activity was inhibited. Meanwhile, *Staphylococcus aureus* MRSA was found to be more resistant to the released compounds, being inhibited by only 44.7% (*i.e.* less than 50% of their activity). Furthermore, the efficiency of the loaded material with silver NPs alone was also included, for which moderate activity was observed against all tested bacteria. In fact, the antibacterial sensitivity of the potent materials increased the inhibition process towards bacterial pathogens, especially for a high concentration of the loaded composite. In addition, facilitated diffusion of the loaded compounds through the tested cotton gauze played an important role in the increase of their responses towards the bacterial pathogens, which in turn contributed to the eradication process and increased the effectiveness of the target molecules inside the bacterial cells. The inhibition process of the bacterial pathogens in the case of starter molecules was also observed, particularly against Gram-positive bacteria. Overall, the release of the target composites from the cotton gauze materials definitely contributed to the inhibition process. Therefore, the diffusion rate of the activated compounds from cotton gauze contributed to protection from bacterial contamination during surgery. Our findings confirmed that the loaded compounds at high concentration were correlated with the increases in the inhibition ratio for all bacterial pathogens. Thus, our results showed that the cotton gauze material loaded with a high concentration of target compounds was able to prevent more bacterial proliferation than lower concentrations. Therefore, different concentrations of starter composite-loaded materials were consequently applied to determine the minimum inhibition concentration (MIC).

**Table 1:** Inhibition in bacterial survival (%) after treatment with the target materials using the colony-forming unit (CFU) method

Sample no. final codes	Inhibition in bacterial survival, %		
	*Staphylococcus aureus* MRSA	*Bacillus cereus*	*Pseudomonas aeruginosa*
Cotton gauze with (AgNPs/gel)	4.7 ± 1.33	9.5 ± 2.21	ND
Cotton gauze with (AgNPs/SBPBTT^1^/gel)	13.5 ± 3.15	34.9 ± 4.55	18 ± 2.55
Cotton gauze with (AgNPs/SBPBTT^2^/gel)	44.7 ± 4.22	72.4 ± 5.15	57.6 ± 3.55
Cotton gauze with AgNPs	31.3 ± 2.85	56.9 ± 1.72	46.9 ± 1.11

**Starter compounds**			
SBPBTT	41.2 ± 1.25	62.4 ± 3.35	39.8 ± 4.22
Cotton gauze with SBPBTT	52.9 ± 1.25	40.6 ± 2.25	29.8 ± 2.25
Cephradine^a^	ND	ND	ND
Ampicillin^a^	ND	ND	ND
Ciprofloxacin^a^	53.7 ± 5.55	67.3 ± 4.5	48.8 ± 1.8
Erythromycin^a^	ND	ND	ND
Polymyxin b^a^	ND	ND	36.3 ± 1.08
Kanamycin^a^	27.8 ± 3.2	23.9 ± 1.55	ND

a Cephradine, ampicillin, ciprofloxacin, erythromycin, polymyxin b and kanamycin were used as standard antibacterial agents at 20 µg mL^−1^. ND: not determined.

### 3.4 Determination of the MIC value of the target composite

The response of the starter molecules that were incorporated into the cotton gauze materials with different concentrations toward the bacterial pathogens should be confirmed *via* the value of the minimum inhibition concentration (MIC). Therefore, the utilization of different concentrations for both starter compounds was investigated, as listed in Table 2.

**Table 2:** Minimum inhibitory concentration (MIC) of the starter compounds

Sample no. final codes	Minimum inhibitory concentration (MIC, µg ml^−1^)		
	*Staphylococcus aureus* MRSA	*Bacillus cereus*	*Pseudomonas aeruginosa*
**Starter compounds**			
SBPBTT	40 ± 5.25	10 ± 2.25	10 ± 2.12
Cotton gauze with SBPBTT	20 ± 1.55	10 ± 3.33	40 ± 6.66
Cephradine^a^	>60	>60	>60
Ampicillin^a^	>60	>60	>60
Ciprofloxacin^a^	20 ± 2.55	10 ± 1.5	20 ± 2.8
Erythromycin^a^	>60	>60	>60
Polymyxin b^a^	>60	>60	30 ± 4.22
Kanamycin^a^	40 ± 3.2	20 ± 2.15	>60

a Cephradine, ampicillin, ciprofloxacin, erythromycin, polymyxin b and kanamycin were used as standard antibacterial agents.

A potential induced anti-proliferative effect of the cotton gauze could be based on two factors: first, the concentration of the loaded starter compounds; second, the diffusion rate of the compounds through the cotton materials. Thus, the concentration of the loaded compounds is a critical factor that is observed to have a significant effect against bacterial pathogens. In addition, increases in the surface area of the cotton material have also been shown to have a significant effect on bacterial pathogens due to increases in the active molecules released. In this respect, a lower MIC value was found in the case of *Bacillus cereus*, followed by *Pseudomonas aeruginosa* and *Staphylococcus aureus* (Table 2). In this context, the ability of the target compounds to penetrate bacterial pathogens clearly reflected their potential to disrupt them at a certain concentration. This result could be discussed in terms of the contribution of the MIC value to inhibition of bacterial pathogens.

### 3.5 ROS quantification inside the treated bacterial cells

The effectiveness of the target materials towards bacterial pathogens, in particular those loaded at a high concentration of the active compounds, may be a result of the reactive oxygen species (ROS) mechanism. Potential generation of ROS from the Ag NPs may contribute to penetration of the bacterial cell wall, causing disruption. Therefore, after incubation of the bacterial pathogens in the presence of the target materials at different concentrations, each sample of treated cells was washed three times with PBS buffer and centrifuged at 8000 rpm for 12 min. The cell-free extract was mixed with the fluorescence probe, DCFH, for 30 min in the dark, and the fluorescent intensity of the formed DCF was measured using spectrofluorometry, as outlined in the materials and methods section.

As can be seen in Table 3, lower activity of the generated ROS from the target materials was indicated. However, some ROS released from the starting molecules was detected, such as for sample SBPBTT against Gram-positive bacteria, *Bacillus cereus* > *Staphylococcus aureus*. Furthermore, a lower level of ROS was generated inside the Gram-negative bacterium, *Pseudomonas aeruginosa*, which showed minimal increases in fluorescence intensity (21.9%, 3.99% for SBPTE and SBPBTT compounds, respectively) compared to the untreated cells. In fact, no intracellular increases in ROS in the target materials and starting compounds were observed compared to the standard drug (ciprofloxacin). In this respect, we can conclude that the ROS factor does not support the mechanism of cell disruption by the tested materials. As suggested by,^46^ excessive ROS generation inside bacterial cells during apoptosis, resulting from oxidative stress, contributes to cell death, whereas moderate or lower ROS levels are generally considered part of the cells' physiological defense mechanisms.^47^

**Table 3:** Quantification of ROS inside the bacterial pathogens after treatment

Sample no. final codes	ROS determination (DCF level, counts)		
	Using spectrofluorometry (excitation and emission wavelengths at 485 nm and 530 nm, respectively)		
	*Staphylococcus aureus* MRSA	*Bacillus cereus*	*Pseudomonas aeruginosa*
Cotton gauze with (AgNPs/gel)	96.8 ± 2.22	103.8 ± 3.81	93.6 ± 1.61
Cotton gauze with (AgNPs/SBPBTT^1^/gel)	119.1 ± 3.15	114.9 ± 2.77	87.6 ± 2.35
Cotton gauze with (AgNPs/SBPBTT^2^/gel)	111.2 ± 2.12	122.4 ± 4.45	108.6 ± 3.05

**Starter compounds**			
SBPBTT	141.8 ± 4.05	166.4 ± 5.22	128.2 ± 3.02
Cotton gauze with SBPBTT	129.5 ± 3.15	120.4 ± 3.32	109.3 ± 1.81
Untreated cells	99.5 ± 4.27	109.8 ± 2.99	105.1 ± 2.91
Cephradine^a^	ND	ND	ND
Ampicillin^a^	ND	ND	ND
Ciprofloxacin^a^	402 ± 11.25	382.3 ± 8.52	478.8 ± 9.22
Erythromycin^a^	ND	ND	ND
Polymyxin b^a^	ND	ND	ND
Kanamycin^a^	112 ± 9.2	93.9 ± 1.55	ND

a Standard antibiotics used in this experiment.

### 3.6 Effect of the target materials on bacterial lipid peroxidation (LPO)

The difficulty of penetrating the bacterial cytoplasmic membrane is considered an important measure of inhibition by the tested treatments, demonstrating their ability to contribute to bacterial eradication. In this, oxidative stress on the bacterial cell wall usually triggers oxidation of the fatty acid content of the cell wall, resulting in easy penetration of the tested compound inside the bacterial cell. The level of the obtained lipid peroxidation refers to successful treatment to overcome the complicated cell wall. Therefore, an LPO test was undertaken to evaluate the oxidation potential of bacterial cell membrane fatty acids by the target cotton gauze and its loaded compounds. Accordingly, treatments of each tested bacterial strain in the presence of cotton gauze and starter compounds under normalized conditions were investigated (Table 4).

**Table 4:** Lipid peroxidation activity

Sample no. final codes	Lipid peroxidation efficiency (%)		
	*Staphylococcus aureus* MRSA	*Bacillus cereus*	*Pseudomonas aeruginosa*
Cotton gauze with (AgNPs/gel)	77.3 ± 4.25	49.7 ± 1.81	29.5 ± 3.21
Cotton gauze with (AgNPs/SBPBTT^1^/gel)	119.7 ± 2.05	154.9 ± 8.15	87.2 ± 5.55
Cotton gauze with (AgNPs/SBPBTT^2^/gel)	204.2 ± 7.02	252.4 ± 5.15	157.6 ± 2.25

**Starter compounds**			
SBPBTT	177.2 ± 1.25	232.4 ± 3.35	129.8 ± 4.22
Cotton gauze with SBPBTT	222.9 ± 1.25	210.6 ± 2.25	169.8 ± 2.25
Cephradine^a^	ND	ND	ND
Ampicillin^a^	ND	ND	ND
Ciprofloxacin^a^	362 ± 7.05	412.3 ± 2.55	278.8 ± 8.8
Erythromycin^a^	ND	ND	ND
Polymyxin b^a^	ND	ND	ND
Kanamycin^a^	112 ± 9.2	93.9 ± 1.55	ND

a Standard antibiotics used in this experiment.

In this respect, there were significant differences in lipid peroxidation activity by the cotton-gauze-loaded materials and the starter compounds. A maximum level of fatty acid oxidation was notably obtained by the potent compound 3 (SBPBTT) against all bacterial pathogens, while the starter compounds proved to be more active for lipid peroxidation toward bacterial pathogens. Distinctive oxidation of fatty acid content for pathogens when treated with cotton gauze loaded with a high concentration of target compounds may be related to the lipid peroxidation activity that is triggered by the easy penetration of target compounds inside the bacterial cells. Our findings also showed differences between Gram-positive and Gram-negative bacteria in terms of the cell wall. The rigid cell wall of *Pseudomonas aeruginosa* makes it challenging for the target compounds to diffuse compared to *Bacillus cereus.* The differences in the disruption of the cell wall between the two bacterial types could be discussed in terms of the differences between them in cell wall composition. The bacterial cell wall serves as the first line of defense against external factors and hostile environments. Both Gram-positive and Gram-negative bacteria have layers of peptidoglycan, but Gram-negative bacteria also have an outer membrane composed of lipopolysaccharide. There are clear differences in fatty acid oxidation between Gram-positive and Gram-negative bacteria, where Gram-negative bacteria possess a higher lipid content concentration than Gram-positive ones, along with the presence of the lipopolysaccharide, which also plays an important role in the resistance of Gram-negative bacteria toward fatty acid oxidation. Accordingly, a low fatty acid content in *Bacillus cereus* and *Staphylococcus aureus* facilitated interaction with their fatty acid content and led to greater cell disintegration than that caused in the case of *Pseudomonas aeruginosa*, reflecting a difference in the oxidative stress based on bacterial cell membrane type. Conversely, the starter molecules showed significant LP activity towards the cell membrane of bacterial pathogens, particularly in the case of the SBPBTT with cotton Gauze sample. Collectively, our findings clearly showed that the target molecules could increase the oxidative stress on the bacterial cell wall *via* oxidation of the fatty acid content, especially in the case of the most active compound 3. The standard antibacterial agent also successfully confirmed LP activity towards bacterial pathogens; LP efficiency for ciprofloxacin reached 422% and 335% for *Bacillus cereus* and *Pseudomonas aeruginosa*, respectively.

### 3.7 Water uptake and weight loss

Gauze and other conventional wound dressings serve as a passive barrier. They do not actively interact with the wound bed, but they do absorb exudate. Furthermore, biointeractivity is a feature of biodegradable wound dressings. As they degrade, they release active molecules (*e.g.*, growth factors, antimicrobials) directly to the wound site in a controlled manner.^48^ In fact, the body's initial reaction to injury is what defines the inflammatory phase of the wound–healing process, which lasts for around one to five days: immune cell inflow and hemostasis (clotting) to combat infection and remove debris.^49^ Preventing bacteria from moving from simple contamination (the presence of non-replicating bacteria) to colonization (replicating bacteria) and ultimately to crucial colonization and local infection is the aim of early antimicrobial intervention during the inflammatory phase. It is considerably more difficult to remove a strong biofilm—a structured community of bacteria—once it has formed, and it can seriously hinder the healing process. Thus, this study evaluated the ability of AgNPs-treated cotton gauze to absorb exudate and release active ingredients at the wound bed by monitoring its water uptake and degradation potential over a range of time periods. As shown in Table 5, the untreated cotton showed a consistent and moderate water uptake of around 147–150%. This is the baseline hydrophilic nature of cotton fabrics. Both AgNPs-treated samples were significantly more hydrophilic than untreated cotton, especially on the first day, with water uptake values around 200% (meaning they absorbed an amount of water equal to their own weight). The water uptake for the AgNPs-treated samples decreased sharply over the three days. This is likely a direct result of the biodegradation recorded in the left columns. As the material degrades and loses mass, its structure changes, potentially reducing its ability to retain water in a measurable way. In the same context, the untreated cotton showed no measurable weight loss over 6 d. This indicates high resistance to biodegradation under the tested conditions. While both AgNPs-treated samples showed significant and variable weight loss from the first day, indicating that the coating (AgNPs/SBPBTT^1^/gel) made the cotton fabric much more susceptible to degradation. The two treated samples show different degradation patterns. The first degrades very quickly on day 1 (18.3% loss), while the second shows a more gradual loss, but both end with a similar total loss after 6 d (∼5–11%). This suggests possible variations in the coating process or composition.

**Table 5:** The change in water uptake and weight loss percentage of different cotton gauze fabrics

Sample name	Water uptake (%)			Weight loss (%)					
	1 d	2 d	3 d	1 d	2 d	3 d	4 d	5 d	6 d
Untreated cotton	147.9	149.8	147.7	0	0	0	0	0	0
Cotton gauze with (AgNPs/SBPBTT^1^/gel)	200.8	189.9	151.8	18.3	6.9	11.9	4.5	2	5.8
Cotton gauze with (AgNPs/SBPBTT^1^/gel)	198.4	173.9	144.8	12.7	8.4	4.8	3.9	4.3	10.8

However, contact-killing dressing materials are highly recommended for long-lasting antibacterial activity without depletion and to eliminate cytotoxicity concerns resulting from released agents.^50^ The antibacterial agent release is an alternative approach to create a protective antimicrobial environment in the wound bed during the critical inflammatory phase. This can help “shorten the inflammation phase” by systemically reducing the bacterial load in the entire wound area.^51^

### 3.8 Cytotoxicity evaluation

IC_50_ is the concentration of a substance that causes 50% inhibition of cell viability. The half-maximal inhibitory concentration (IC_50_) in pharmacological research is a factor utilized to evaluate antagonist drug potency, and the effect of dose–response curve of different concentrations of drug on fibroblast cells growth can be used to calculate the IC_50_ (Table 6).

**Table 6:** Cytotoxicity evaluation using concentrations 50, 25, 10 µg of the newly as-prepared different samples against fibroblast cells and calculation of the equivalent concentration IC_50_ for each^a^

Sample	Mean conc. 10 µg	Mean conc. 25 µg	Mean conc. 50 µg	Equation	Mean control	Control IC_50_	Conc. equivalent to IC_50_ µg
SBPBTT	0.685	0.186	0.671	*y* = 14.058*x* + 46.108	0.693	0.3465	50.97
Cotton gauze with SBPBTT	0.265	0.152	0.677	*y* = −114.44*x* + 95.066	0.547	0.2735	126.2
Cotton gauze with AgNPs	0.119	0.28	0.187	*y* = −266.62*x* + 105.41	0.477	0.2385	41.82
Cotton gauze with (AgNPs/gel)	0.591	0.103	0.078	*y* = 142.59*x* + 16.641	0.538	0.269	54.95
Cotton gauze with (AgNPs/SBPBTT^1^/gel)	0.412	0.564	0.967	*y* = −149.27*x* + 150.01	0.642	0.321	102
Cotton gauze with (AgNPs/SBPBTT^2^/gel)	0.953	1.641	2.341	*y* = −64.819*x* + 159.96	0.57	0.285	141.48

a *y* is the concentration equivalent to IC_50_ per µg and *x* is the control IC_50_ absorbance at a wavelength of 630 nm.

The criteria used to categorize the cytotoxicity of isolated fractions against fibroblast cells, based on U.S. National Cancer Institute (NCI) and the Geran protocol,^52,53^ was as follows: IC_50_ ≤ 20 µg mL^−1^ = highly cytotoxic, IC_50_ 21–100 µg mL^−1^ = moderately cytotoxic, IC_50_ 101–200 µg mL^−1^ = weakly cytotoxic and IC_50_ > 501 µg mL^−1^ = no cytotoxicity. The synthesized composites were evaluated for their *in vitro* cytotoxic potential against fibroblast cells. Three concentrations, 50 µg mL^−1^, 25 µg mL^−1^ and 10 µg mL^−1^, of newly prepared composites were used to determine the cytotoxic effect on fibroblast cells compared with the control free of tested samples.

In the present study, the results revealed that the as-prepared tested samples, SBPBTT, cotton gauze with AgNPs and cotton gauze with (AgNPs/gel) showed a moderate cytotoxicity, while the as-prepared tested samples of cotton gauze with SBPBTT, cotton gauze with AgNPs/SBPBTT^1^/gel and cotton gauze with AgNPs/SBPBTT^2^/gel exhibited weak cytotoxicity. These findings strongly suggest that the tested samples are biocompatible and safe within the tested range. The absence of toxicity in fibroblasts is particularly important, as these cells play a critical role in tissue homeostasis and wound repair, supporting the potential therapeutic applicability of these newly synthesized composites without adverse cellular effects.

## 4 Conclusion

AgNPs/gel colloidal solutions were synthesized through a green synthesis route and integrated into cotton gauze and loaded with a newly synthesized bis-compound to advance the wound-healing process. Surface chemical analysis by XPS revealed the presence of metallic silver (Ag^0^) within the composite coating, with the Ag 3d spectrum showing definitive peaks at 368.0 eV and 374.0 eV, confirming the successful synthesis of silver nanoparticles on the cotton gauze. A notable antibacterial activity of cotton-gauze-loaded AgNPs/gel could be efficiently related to higher level of the lipid peroxidation activity in the bacterial cell wall. In addition, the higher concentration of the AgNPs/gel, along with regulation of the release rate, was directly proportional to the inhibition of the bacterial pathogen proliferation. Overall, this study shows that cotton-treated fabrics offer a viable antibacterial wound dressing for applications involving the healing of infected wounds.

## 5 Recommendations for future work

Herein, cotton fabrics were functionalized with AgNPs/SBPBTT/gel to target bacterial populations that could otherwise impede wound healing. Additional research and development should optimize the chemical and physical stability of the fabricated samples under physiological environments to determine mechanical strength, water uptake capacity and biodegradation behavior. The sustained release of the bioactive agents AgNPs and SBPBTT at different pH levels should be studied. Further biological analyses should be undertaken, including fibroblast proliferation and migration. Conducting an *in vivo* animal model is essential to determine the biocompatibility and safety profile of AgNPs/SBPBTT/gel for the development of more effective clinical strategies for wound management and care.

## Conflicts of interest

All the authors have no conflict of interest.

## Data Availability

All the data used are included in the manuscript.
